# Immunomodulatory efficacy of culture condition-dependent modulation of mesenchymal stem cells

**DOI:** 10.1186/s13287-026-05169-0

**Published:** 2026-07-13

**Authors:** Qiuli Sun, Xi Zhang, Huixian Dong, Chunliu Mi, Shaolei Geng, Qin Li, Jihong Zhang, Xiaoyin Wang, Tianyun Wang

**Affiliations:** 1 School of Life Science and Technology, Henan Medical University, Xinxiang, 453003 China; 2 International Joint Research Laboratory for Recombinant Pharmaceutical Protein Expression System of Henan, Henan Medical University, Xinxiang, 453003 China; 3 Henan Key Laboratory of Neurorestoratology and Protein Modification, The First Affiliated Hospital of Henan Medical University, Xinxiang, 453003 China; 4 School of Pharmacy, Henan Medical University, Xinxiang, 453003 China; 5 School of Basic Medicine, Henan Medical University, Jinsui Road, Xinxiang, 453003 Henan China

**Keywords:** Chemical additives, Culture environment, Cytokines, Immune regulation, Mesenchymal stem cells

## Abstract

Mesenchymal stem cells (MSCs), due to their unique anti-inflammatory, immunosuppressive, immunomodulatory, and regenerative properties, have been used in the treatment of various diseases. Despite the advancements in the technological development of culture media and cell cultures, ensuring that MSCs have immune regulatory functions while maintaining their application remains challenging. In this review, factors that affect the proliferation, stemness, immune regulation, and paracrine properties of MSCs in culture are summarized, and divided into three groups: culture environment, cytokines, and chemical additives. Understanding the factors that affect the immune regulatory properties of MSCs in culture will contribute to the large-scale cultivation of MSCs, in order to obtain high-quality MSCs for disease treatment.

## Introduction

**Fig. 1:**
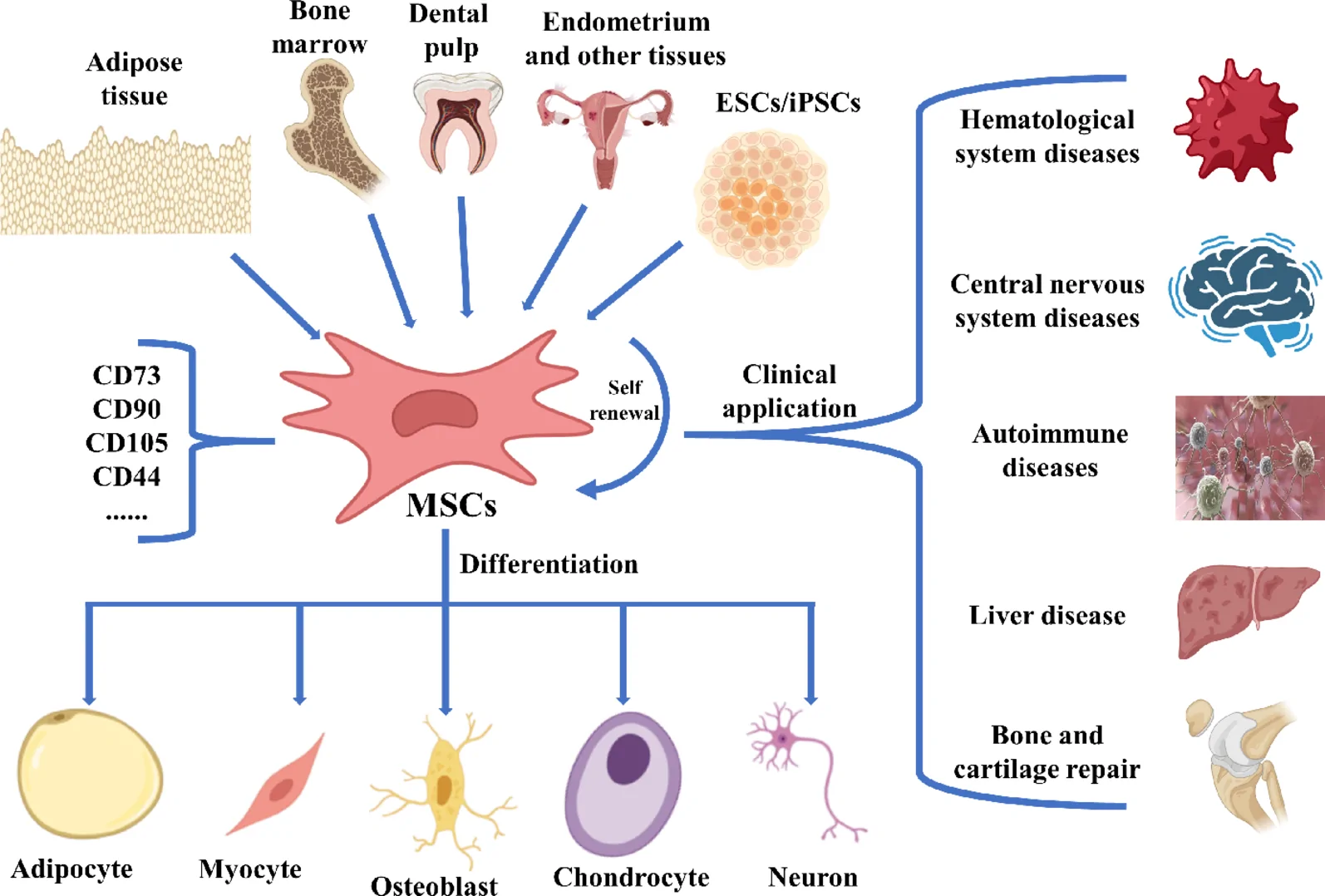
Common sources of MSCs, characterizing cell surface markers, potential cell types after differentiation, and potential target organs for stem cell therapy [20]

Stem cells are a special type of cells with the potential for self-renewal and multi-directional differentiation. They can divide to produce more stem cells and differentiate into various cells with specific functions in the body, serving as the foundation for tissue repair, regeneration and development. At present, stem cells mainly include three types: embryonic stem cells (ESCs), induced pluripotent stem cells (iPSCs), and adult mesenchymal stem cells (MSCs). Numerous studies have reported that ESCs, iPSCs, and MSCs can be applied in the treatment of various diseases and drug screening [1–8]. However, the ethical concerns of ESCs [9] and the potential risks of gene editing safety and tumorigenicity of iPSCs [10, 11] limit their clinical applications. MSCs are widely available, including bone marrow, umbilical cord, placenta, and adipose tissue [12–14], and possess multi-directional differentiation potential, low immunogenicity, and immune regulatory advantages [15], making them an ideal starting material for tissue engineering [16], cell reprogramming [17–19], and cell trans-differentiation (Fig. 1).

In 1999, scientists first demonstrated that MSCs have the ability to differentiate into adipocytes, osteoblasts, chondrocytes, and other cells in multiple directions [21]. MSCs, as important adult stem cells in regenerative medicine, possess regenerative properties such as self-renewal, secretion of nutritional factors, and the ability to induce mesenchymal cell lineages. They can also regulate the immune system and enhance tissue repair. Therefore, it has broad application prospects in cell therapy [22, 23]. In recent years, clinical researches on MSCs have developed rapidly and achieved significant effects. For many diseases such as Alzheimer’s disease [24, 25] and amyotrophic lateral sclerosis (ALS) [26, 27], MSCs are expected to represent a promising therapeutic approach [28–30]. With the advancement of technology and the development of the industry, the research and development of MSC drugs involves an increasingly wide range of areas, and the types of diseases targeted are also more diverse. As of February 4, 2024, the Drug Evaluation Center of the National Medical Products Administration of China has given great attention and support to clinical trials of MSCs, with a total of 53 clinical trials implicitly approved, covering 46 indications (The information is sourced from stem cell and genetic science). It is worth mentioning that the stem cell materials used in these clinical trials mainly come from umbilical cord, placenta, bone marrow, dental pulp, uterine blood, fat, etc. Among them, there are 33 items mainly based on umbilical cord. These diseases encompass a range of related conditions that seriously affect people’s quality of life, such as Alzheimer’s disease, ALS, chronic heart failure caused by chronic ischemic cardiomyopathy, chronic obstructive pulmonary disease, acute respiratory distress syndrome (ARDS), stroke, liver failure, periodontal disease, rheumatoid arthritis, diabetes foot ulcer, traumatic spinal cord injury, etc [24–27, 31]. According to a report released by Analytical Research Cognizance, the global MSCs market is rapidly developing, with a compound annual growth rate of 4.3% from 2020 to 2025, and is expected to reach a market size of 230 million US dollars by the end of 2025. The report points out that in the coming years, the Asia Pacific region will occupy more market share, especially China.

In order to improve the efficiency of its application in the fiercely competitive MSC market, it is necessary to enhance the culture quality of MSCs, such as their ability to self-renewal, secrete nutritional factors, and induce mesenchymal cell lineages. The self-renewal ability is directly related to the “stemness” of cells. High-stem MSCs have higher multi-directional differentiation potential and secretory activity, which is the basis for them to exert therapeutic effects [32, 33]. The main mechanism of the clinical application of MSCs is not to differentiate into target tissue cells and directly replace them, but to achieve effects such as immune regulation, anti-fibrosis, anti-apoptosis and pro-angiogenesis through their powerful paracrine effect [34]. With the advancement of culture media and cell culture techniques, researchers have been striving to optimize the culture conditions of MSCs to improve their proliferation rate and stability of stem cell characteristics [35–37]. By adding growth factors, extracellular matrix components, or regulatory factors, the survival, differentiation, and function of MSCs can be improved [38–40]. However, maintaining the high proliferation rate of MSCs while preserving their functionality remains challenging. Therefore, the current emphasis on developing cell culture processes has transitioned from merely attaining high-yield cells to ensuring the consistent production of high-quality cells that are effectively utilized. High quality MSCs refer to their ability to maintain their own stem cell characteristics, such as self-renewal, secretion of nutritional factors, and induction of mesenchymal cell lineages. It is crucial to comprehend the factors that influence the quality attributes in cell culture when producing therapeutic stem cells with consistently high quality.

In this review, we will discuss the key quality attributes of MSCs, including proliferation ability, stemness markers, multipotent differentiation potential, and paracrine activity. Additionally, we will explore the factors that influence these attributes during cell culture, such as the culture environment, cytokines, and chemical additives.

## Immunotherapy attributes of MSCs

The efficacy of MSCs immunotherapy is partly dependent on the quality of the cultured stem cells. To assess the quality of the desired MSCs, it is critical to identify the quality attributes that impact their proliferation and functionality during MSC cultivation. The key quality attributes of MSCs typically comprise their proliferation, differentiation, and paracrine activities.

### Proliferation ability

MSCs have the potential for self-replication and multi-directional differentiation, which means they can proliferate and differentiate into multiple types of cells. If the proliferative capacity of stem cells is compromised, their ability to proliferate effectively diminishes, which can severely impact subsequent functions and applications. This includes a reduction in tissue repair and regeneration capabilities, dysregulation of immune function, heightened vulnerability to diseases, deterioration of overall health status, and diminished efficiency of stem cell therapies [41–43]. Therefore, preserving the normal proliferative function of stem cells is crucial for maintaining human health and preventing disease.

Telomeres are repetitive sequences of nucleotides placed at the end of each chromosome to prevent their destruction and adherence to neighboring chromosomes. Telomere length is associated to proliferative capacity, and its shortening is caused by terminal replication problems [44]. The shortening of telomeres is also related to the aging of cells, and when the telomeres are shortened to a certain extent, the cell can no longer divide and enter a dormant or dead state. In MSCs, long-term culture leads to replicative senescence of the cells, which is manifested by changes in cell morphology, migration and differentiation potential, as well as reduced cytoskeleton renewal and impaired mitochondrial morphology. These changes increase susceptibility to cellular senescence. Fibroblast growth factor 2 (FGF2) enhances telomerase activity and increases telomere length in hESC via the Wnt/β-catenin signaling pathway [45]. Supplementing an FGF2-binding heparan sulfate variant (50 mg/mL) helps preserve the properties of stem cells [46]. The length of telomeres may be a factor in regulating aging, as their length decreases with age [45]. Heparin interacts with and activates a variety of mitogenic factors and morphogens that facilitate the proliferation and lineage commitment of progenitor cells. Previous studies have reported that heparin accelerates the proliferation of genetically modified hMSCs [47]. Furthermore, heparin supplementation has been shown to enhance hMSC proliferation without altering the expression levels of stem cell markers. However, it is important to note that high doses of heparin significantly inhibit hMSC growth, suggesting that heparin supplementation in culture may compromise cell fate decisions [48].

### Stemness markers

To classify MSCs, the expression of surface markers is a critical requirement as outlined by the International Society for Cell and Gene Therapy (ISCT). More than 95% of MSC populations must express CD73, CD90, and CD105, while less than 2% should express CD45, CD34, CD14 or CD11b, as well as either CD79a or CD19 and HLA class II [49]. Abnormalities of surface marker expression may indicate a heterogeneous cell population or reflect poor cell quality due to low viability, inadequate differentiation capacity, or a stressed or immune-stimulated state.

MSCs possess the capacity for self-renewal and multidirectional differentiation, which are intricately linked to the expression of specific surface markers. For instance, the expression of markers such as CD73, CD90, and CD105 is crucial for maintaining the undifferentiated state of MSCs, thereby enabling their continued proliferation and differentiation [50, 51]. MSCs play a pivotal role in tissue repair and regeneration. Their surface markers, such as CD44, are integral to the processes of cell migration and adhesion, facilitating the movement of MSCs to sites of injury where they can differentiate into specific cell types, thereby contributing to the repair and regeneration of damaged tissues [52, 53]. The communication and interaction between MSCs and other cellular entities also hinge on the expression of these surface markers. For instance, through adhesion molecules like CD54, MSCs can engage with various immune cells and endothelial cells, participating actively in the regulation of inflammation and immune responses [54].

In summary, there exists a profound relationship between the surface markers of MSCs and their functional roles. These markers not only serve as identifiers for isolating MSCs but also reflect their biological characteristics and functional states [55, 56]. By further investigating the regulatory mechanisms governing the expression of these markers along with their implications for cellular function, we can develop more precise and effective strategies for clinical applications involving MSCs.

### Multipotent differentiation potential

Recent advancements in MSCs technology have opened new avenues for patients suffering from diseases and disorders that remain untreated. Stem cell-based therapies utilizing multipotent MSCs have emerged as pivotal components in regenerative medicine [57]. According to ISCT guidelines, MSCs are multipotent progenitor cells with self-renewal capabilities (limited in vitro) and the potential to differentiate into various mesenchymal lineages such as osteoblasts, chondrocytes, and adipocytes. The multidirectional differentiation capability of MSCs positions them as pivotal agents in the treatment of various diseases. Adipose-derived stem cells (ASCs), recognized for their potential in cellular therapies, offer several advantages, including a readily available tissue source for isolation, straightforward methodologies for tissue collection and cell extraction, and significant therapeutic potential. Research conducted both in vitro and in vivo has established that ASCs are multipotent; they possess the ability to differentiate into various cell types originating from the mesoderm, such as adipocytes, chondrocytes, osteoblasts, among others. As of November 2021, data retrieved from clinicaltrials.gov (accessed on November 28, 2021) indicated that there were 440 clinical trials assessing the efficacy of ASCs in treating a range of diseases and disorders-including osteoarthritis, cardiovascular disease, multiple sclerosis, inflammatory bowel disease (IBD)/Crohn’s disease-and more [58].

One of the significant challenges in utilizing MSCs for regenerative medicine and tissue engineering is their propensity to undergo spontaneous differentiation during prolonged in vitro expansion [59]. MSCs isolated from different donors exhibit distinct differentiation capacities. Through single-cell multiomics analysis, Chen et al. investigated the transcriptome and epigenome of MSCs derived from four healthy donors, revealing that these cells possess varying chromatin-accessible regulatory elements that govern gene expression [60]. Furthermore, a study examining the in vitro differentiation of human MSCs into osteogenic, chondrogenic, fibroblastic, and adipogenic lineages identified 148 genes-nine of which encode transcription factors-that are significantly upregulated (more than two-fold compared to mature cells) [61]. The molecular genetic mechanisms, signaling pathways, factors, and markers associated with the differentiation of MSCs into osteogenic, chondrogenic, fibroblastic, and adipogenic lineages have been actively researched within the domain of cell technology.

### Paracrine activities

The primary mechanism underlying MSC-based therapies is their paracrine function, which encompasses the secretion of a diverse array of soluble factors that exert immunomodulatory, angiogenic, anti-apoptotic, and antioxidative effects [62]. MSCs, as feeder cells, can secrete various factors that promote mitosis, such as bFGF, epidermal growth factor (EGF), and β-nerve growth factor (NGF). As a feeder layer, MSCs can provide a complex microenvironment between cells. For instance, Di Nicola et al. demonstrated that soluble factors secreted by MSCs significantly contribute to their immunomodulatory effects when co-cultured with T-lymphocytes using a Trans-well system [63]. Currently, ASCs have garnered considerable attention as promising candidates for cellular therapies. These cells release a wide range of cytokines, growth factors, nucleic acids (including microRNAs), and other macromolecules into the surrounding environment through both direct secretion and microvesicle-mediated transport. The secretome of ASCs has been shown to influence tissue biology, stimulate resident stem cells within tissues, modulate immune cell activity, and mediate therapeutic outcomes effectively [58].

The trophic factors secreted by MSCs may be partially attributed to the release of paracrine factors, including exosomes. MSC-derived exosomes are abundant in various proliferative and anti-apoptotic factors, as well as a range of chemokines [64]. Studies have demonstrated that MSC exosomes possess certain immunomodulatory effects; furthermore, MSCs migrate to injury sites to mitigate inflammation by inhibiting inflammatory mediators and chemokines. MSCs can also exert immunomodulatory effects through paracrine mechanisms or direct cell-to-cell contact. Zhang et al. discovered that MSC-derived exosomes promote the proliferation, migration, and angiogenic differentiation of endothelial progenitor cells in rat skull defect repair models. Notably, their exosome miR-21 serves as a potential intercellular messenger that enhances angiogenesis via upregulation of the NOTCH1/DLL4 signaling pathway [65]. Additionally, MSCs can influence fibroblasts and keratinocytes through paracrine signaling, thereby creating a biological microenvironment conducive to healing and promoting fibroblast proliferation [66]. This process not only reduces fibrosis but also improves cellular vitality at the site of injury.

## Factors affecting immunotherapeutic properties of MSCs during cell culture

**Fig. 2:**
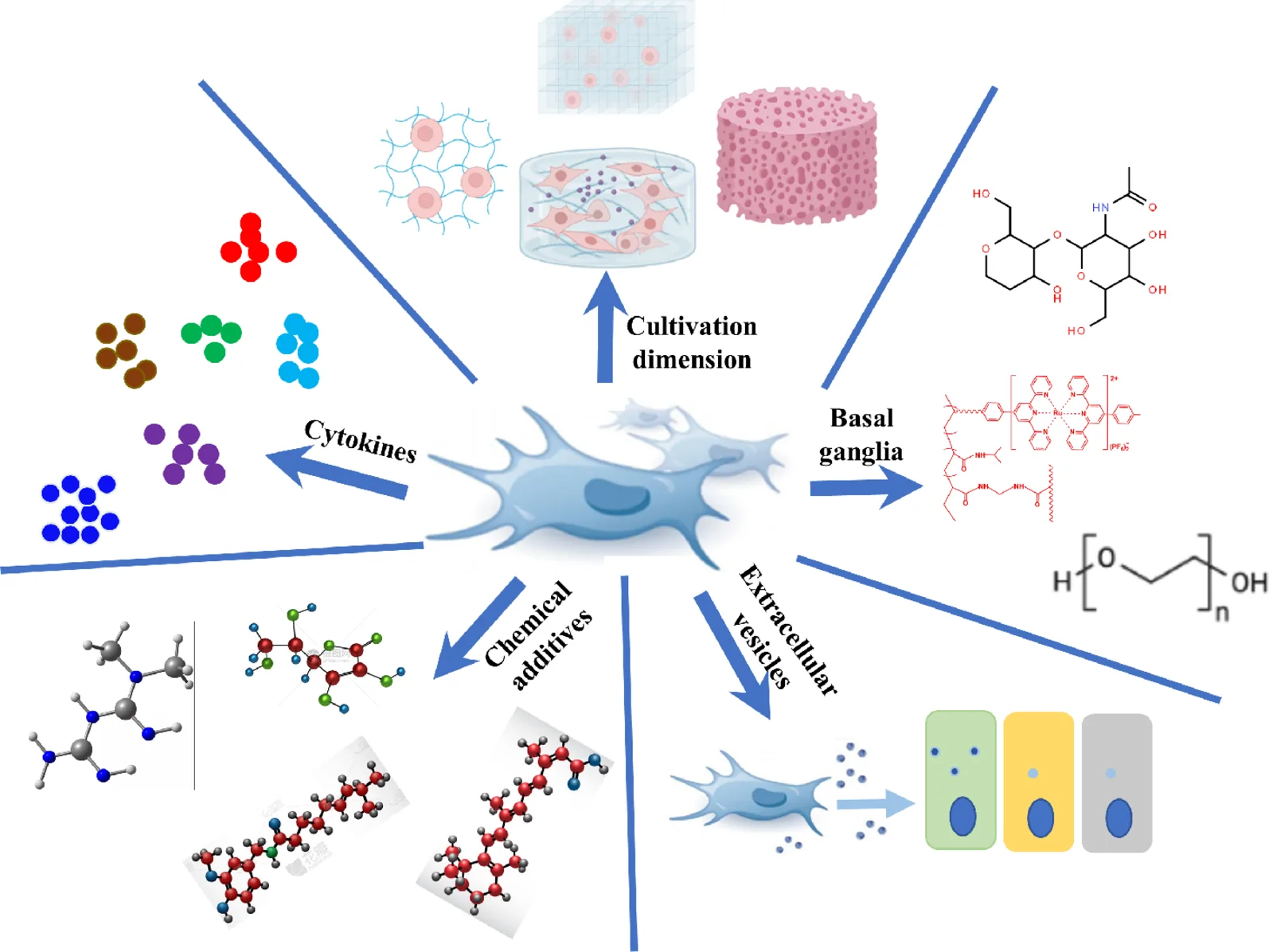
The main factors affecting immunotherapy attributes of MSCs during cell culture include cultivation dimension, basal ganglia, extracellular vesicles, chemical additives, cytokines and etc [20]

The quality of therapeutic MSCs may be influenced by many factors during the culture process, such as cell source and cell culture. The quality of MSCs can change during culture due to several influencing factors. To ensure the desired quality of MSCs is achieved, it is essential to understand these factors throughout the culture process. The primary determinants affecting the therapeutic properties of cultured MSCs include the culture environment itself along with cytokines and additives aimed at enhancing both yield and quality (Fig. 2).

### Culture environment

#### Basal ganglia

In 3D culture environments, various scaffold materials are commonly employed to maintain spatial structure, such as chitosan and hydrogels. Chitin, a long-chain polymer composed of N-acetylglucosamine-a derivative of glucose-serves as biodegradable bioadhesive and biocompatible material that has received approval from the U.S. Food and Drug Administration (FDA) for human applications [82, 83]. Numerous studies have explored the interactions between MSCs and chitin-based materials; one notable example is chitin-based nanoscaffolds. Research indicates that these nanoscaffolds enhance cell proliferation while simultaneously increasing the secretion of paracrine factors. This enhancement promotes angiogenesis and duct formation when co-cultured with human umbilical vein endothelial cells (HUVECs), resulting in greater luminal area, total length, and branching points in tubule formation on chitin-based nanoscaffolds [78]. Hydrogels are highly flexible crosslinked hydrophilic polymers characterized by substantial water content; they provide a versatile platform for MSC encapsulation. Hydrogel-encapsulated MSCs demonstrate improved engraftment capabilities along with enhanced immunomodulatory functions in vivo [84]. 3D hydrogel scaffolds formed through Michael-type addition reaction-by combining two 8-arm PEG polymers functionalized with either thiol (PEG-8-SH) or acrylate end groups (PEG-8-Acr)-create microenvironments conductive to maintaining self-renewal and pluripotency over extended periods. Cells grown with these scaffolds exhibit upregulation of core pluripotency genes including *OCT4*, *NANOG*, and *SOX2*. Interestingly, mechanosensitive genes, *YAP* and *TAZ* also show increased expression levels under these conditions [85]. Co-encapsulation of HUVECs alongside UCMSCs within DN hydrogel facilitates the development of more mature vascular structures compared to hydrogels containing only single cell types both in vitro and in vivo [86]. The encapsulation of MSCs in various hydrogels has emerged as a promising strategy to enhance their immunomodulatory function. Notably, hydrogel parameters should be finely tuning the immunomodulatory functions of the encapsulated MSCs.

#### Cultivation dimension

The stemness of MSCs is maintained within the body’s microenvironment, facilitating not only interactions between cells and the extracellular matrix but also providing essential growth factors. Therefore, the conditions for cell culture are essential for promoting the characteristics of stem cell viability, maintenance, and proliferation [14]. In conventional or traditional two-dimensional cell culture systems, cells grow as monolayers. Although this method is simple to operate and relatively low-cost, it can result in the production of cells with limited ability to differentiate and proliferate and limited stemness properties, for example, fails to mimic the 3D structure found in the human body and poor ECM-cell interactions [67]. Therefore, traditional two-dimensional cell culture has gradually been supplemented and replaced, and researchers tend to use three-dimensional culture systems to simulate conditions closer to the in vivo microenvironment.

3D culture is a culture model in which cells are more similar in shape, adhesion, behavior, and morphology to the in vivo microenvironment. It has interactions between cells, as well as between cells and extracellular matrix, and can better simulate the physiological microenvironment of the human body [68–70]. In order to reduce the limitations of traditional single-layer culture, researchers have gradually developed different 3D culture methods, including cell spheres, hydrogel scaffolds, 3D printing organs, microfluidic chip multicellular, etc. Their respective advantages and disadvantages are shown in Table 1. These 3D models are widely used in disease models and drug screening [71–74]. 3D cell culture is considered a culture model that is more similar to the in vivo microenvironment.

**Table 1:** Advantages and disadvantages of various 3D cultivation methods

Cultivation methods	Advantages	Disadvantages	References
Spheroid culture	1. Facilitate cell-cell and cell-matrix interaction 2. Provide a similar physicochemical environment to the in vivo 3. Maintain intrinsic phenotypic properties 4. Promote the stemness marker expression 5. Secrete cytokines, chemokines and angiogenic factors 6. Improve viability and proliferation	1. Hypoxia and necrosis in the spheroid core 2. Diffusion gradient with increased spheroid size	[14]
Hydrogel scaffolds	1. Vast and tunable properties 2. Enhanced biocompatibility and high hydrophilicity 3. Simulate the internal microenvironment and enhance cell proliferation 4. Facilitate the establishment of drug screening and disease models	1. High cost 2. Complex operation 3. Diffusion restriction 4. Possible cytotoxicity	[75]
3D printing organs	1. Provide a three-dimensional microenvironment 2. Enhance intercellular interactions 3. Personalized customization	1. Cytotoxicity or immune response 2. The types of 3D printed biomaterials are limited 3. Technical challenges 4. High cost	[76]
Microfluidic chip multicellular	1. Simulates the microenvironment in vivo 2. High throughput analysis 3. Precise control of culture conditions 4. Real-time monitoring	1. Technical challenges 2. Uneven distribution of cells 3. Cause cell damage 4. Diffusion restriction	[77]

Under 3D culture conditions, cellular proliferation is enhanced alongside an increase in paracrine factor secretion-such as vascular endothelial growth factor (VEGF), hepatocyte growth factor (HGF), Interleukin-6 (IL-6), and Interleukin-8 (IL-8)-by umbilical cord mesenchymal stem cells (UCMSCs) [78]. Additionally, under 3D culture conditions, the expressions of CD31, CD133 and CD271 of UCMSCs were upregulated, the angiogenesis ability was enhanced in vitro, and the gene expression levels of several hematopoietic factors were upregulated, which helped them play a role in immune regulation [78]. Also, notable upregulation in gene expression levels of *OCT4*, *NANOG*, and *CXCR4*, as well as paracrine factors such as PGE2, TSG-6 and bFGF was observed [79]. 3D culture technology enables cells to touch and interact with each other in three-dimensional space, thereby facilitating information exchange between MSCs and other immune cells. For example, cell-to-cell binding contact via adhesion molecules such as intracellular adhesion molecule (ICAM-1) and vascular cell adhesion molecule (VCAM-1) is a precursor condition for MSCs to exert immunosuppressive effects [80]. In 3D culture environment, such cell-cell interactions may be more frequent and efficient. MSCs may secrete more immunosuppressive factors such as prostaglandin E2 (PGE2), IL-6, and tumor necrosis factor-inducible gene 6 (TSG-6) under 3D culture conditions [81]. These factors play an important role in inhibiting T cell activation and promoting immune tolerance. 3D culture technology provides a more stable and suitable growth environment for MSCs, which helps to improve cell survival and stability. This helps MSCs continue to play a role in immune regulation. By optimizing the 3D culture conditions, the immunomodulatory ability of MSCs can be further improved, providing strong support for their application in the field of disease therapy and cell therapy.

### Cytokines

Cytokines play an essential role as additives in culture media for cultivating MSCs effectively. Cytokines can activate a variety of proliferation-related signaling pathways within MSCs. For example, platelet-derived growth factor (PDGF) can bind to receptors on the surface of MSCs to activate signaling pathways such as PI3K-Akt and MAPK/ERK [87]. Activation of these pathways will promote the expression and activity of cell cycle-related proteins, and promote MSCs from the quiescent phase to the active proliferative phase of the cell cycle. The addition of insulin-like growth factor (IGF) can enhance the uptake and utilization of nutrients such as glucose and amino acids by MSCs, and promote metabolic processes such as intracellular protein synthesis and ATP production, thereby supporting the rapid proliferation of MSCs [88, 89]. Therefore, in the culture of MSCs, adding appropriate concentration of cytokines can significantly improve the proliferation rate of cells, providing sufficient cell sources for subsequent cell therapy or tissue engineering. The addition of some cytokines can inhibit the natural tendency of stem cells to differentiate, thereby maintaining stem cells’ dryness. This is essential for maintaining stem cells’ pluripotency and ability to self-renew. For example, leukemia suppressor factor (LIF) can activate the JAK-STAT3 signaling pathway, and continued activation of this pathway encourages stem cells to remain in an undifferentiated state, preventing them from differentiating into specific cell types [90]. In addition, cytokines such as bFGF can also up-regulate the expression levels of genes associated with dry maintenance, further ensuring that stem cells’ dryness are maintained [91].

HGF can promote both the proliferation and differentiation of MSCs while simultaneously inhibiting their apoptosis, thereby ensuring their survival and functionality. HGF has a broad range of applications in biomedical fields, including tissue engineering, regenerative medicine, and disease treatment. For example, in the treatment of liver diseases, HGF can be used as a potential therapeutic agent to promote liver cell regeneration and repair. bFGF is one of the important components in MSCs culture, especially in the culture of embryonic stem cells. It promotes cell proliferation and differentiation while preserving the undifferentiated state of cells. For example, in skin wound repair, bFGF can promote the proliferation and differentiation of skin cells, thus speeding up the healing process of wounds. VEGF is required for induced pluripotent stem cells (iPSCs) to differentiate into cardiomyocytes. Stem cell factor (SCF) is an essential cytokine for the culture of hematopoietic stem cells (HSCs). Interleukin-family cytokines, such as IL-3, IL-6, IL-33 and LIF, are involved in regulating the various stages of stem cell mobilization and have positive regulatory effects. Colony stimulating factor: It can mobilize stem cells, promote neuronal regeneration and vascular regeneration. Different cytokines can induce MSCs to differentiate into specific cell types. In the culture of MSCs, cytokines such as members of the bone morphogenetic protein (BMP) family can induce MSCs to differentiate into osteoblasts [92]. bFGF and TGF-β can induce MSCs to differentiate into chondrocytes or adipocytes. By regulating the type and concentration of cytokines, the direction and degree of MSCs differentiation can be finely regulated, providing a suitable cell source for tissue engineering and regenerative medicine. Cytokines also play an important role in immune and inflammatory responses. In MSC culture systems, the addition of specific cytokines can regulate the immune response by reducing inflammatory factor release; this aids in restoring immune balance within the body and mitigating unnecessary tissue damage. Such mechanisms are particularly significant for treating autoimmune diseases and preventing transplant rejection.

As a medium additive, cytokines play various roles in the culture of MSCs. They can not only promote cell proliferation, maintain stem cell dryness, induce cell differentiation, but also regulate immune and inflammatory responses. By precisely regulating both the type and concentration of growth factors, it becomes possible to accurately control stem cell growth, proliferation, and differentiation. This capability provides robust support for advancements in tissue engineering, regenerative medicine, and related disciplines.

### Chemical additives

Medium additives offer an effective approach to enhance yield and quality during MSCs culture and can be readily implemented. Medium additives can enhance the immunotherapeutic properties of MSCs through various modulations. As a result, increasing attention has been devoted to the positive impacts of medium additives on the culture of MSCs. Extensive endeavors have been made to optimize different additives (Table 2).

**Table 2:** Chemical additives affecting the quality attributes of therapeutic proteins during MSCs cell culture

Name	Range	Function	References
Metformin	0.3–10 mM	Potentiates immunosuppressant activity and adipogenic differentiation	[93]
Capsaicin	0-250 µM	Modulating a variety of signaling pathways, such as PPARγ, C/EBPα and Notch signaling	[94, 95]
Astaxanthin	16 µM	Direct scavenging of free radicals and modulation of cell signaling	[96]
Vitamin C	0–50 µg/mL	Promotes sheet formation and tissue regeneration by elevating telomerase activity	[97]
Dexamethasone	1–3 µg/L	Enhance stemness and immunomodulatory property	[98]
Heparan sulfate	50 mg/mL	Stabilize FGF2-FGF receptor complexes	[46]
Heparin	< 200 ng/mL	Effect on signaling pathways essential to hMSC growth and differentiation (including the TGFβ/BMP superfamily, FGFs, and Wnts)	[99]
Lithium chloride	1–4 mmol/L	Induce an increased cellular reactive oxidative species (ROS) level and activation of ERK1/2	[100]
Insulin	0–20 µM	Promote the Proliferation by Activating the Akt-Cyclin D1 Axis	[101]
Resveratrol and its analogue V29	5–10 µM	Strongly activates sirtuin 1 enhance the proliferation of MSCs	[102]
Rapamycin	0-0.9 mM	Inhibition of AKT/mTOR signaling	[103]

Antioxidants is a potent compound that effectively traps free radicals and protects various types of cells from oxidative damages. The antioxidant activity of Astaxanthin has been found to be nearly 10 times greater than that of other carotenoids such as canthaxanthin, lutein, zeaxanthin, and β-carotene, and 100 times more effective than α-tocopherol [104]. Research indicates antioxidants may exert a direct antioxidant effect by scavenging reactive oxygen species (ROS) from the environment through the Nrf2-ARE pathway, which is recognized as an important endogenous mechanism capable of alleviating intracellular oxidative stress [105, 106]. Astaxanthin not only protects stem cells as a direct free radical scavenger but also enhances their proliferation and differentiation by mitigating oxidative stress primarily through interactions within redox signaling pathways [96]. Consequently, antioxidants are proposed to be utilized as supplementary agents in MSCs transplantation to minimize the adverse effects of oxidative stress on transplanted cells. Vitamin C (VC), a common nutrient vital to human health, is also a common antioxidant and essential for the synthesis and function of immune system factors. VC is necessary to biosynthesize collagen and extracellular matrix (ECM), as well as to mimic the in vivo physiological environment of MSCs and regulate their proliferation and differentiation. Some studies indicate that VC is capable of inducing telomerase activity, leading to up-regulated expression of ECM and stem cell markers [97, 107]. Capsaicin, a member of the vanilloid family, plays a crucial role in determining the proliferation and fate specification of stem cells by modulating a variety of signaling pathways, such as PPARγ, C/EBPα and Notch signaling [94]. But capsaicin appears to inhibit the growth of preadipocytes or BMSCs and induce apoptosis to reduce proliferation by activating caspase-3 and Bax [95, 108].

## Conclusion and future perspective

With increasing public interest in stem cell immunotherapy, it is essential to understand the relationship between key quality attributes of therapeutic MSCs and the factors influencing them during cell culture to obtain high-quality therapeutic products. Numerous factors affect the quality properties of MSCs culture, such as culture environment, cytokines, and chemical additives. For instance, 3D culture in a culture environment is superior to the original 2D culture. In 3D culture, adding scaffold materials such as chitosan and hydrogel to maintain the spatial structure is more conducive to the culture of MSCs. Cytokines can activate multiple proliferation-related signaling pathways within MSCs, promoting the proliferation of MSCs cells. Medium additives can enhance the immunotherapeutic characteristics of mesenchymal stem cells through various adjustments. These factors do not uniformly impact product quality; thus, it is crucial to elucidate the influence of individual factors on product outcomes carefully. During cultivation processes, multiple interrelated factors simultaneously affect product quality.

In conclusion, comprehending the elements that influence MSC immunotherapy during culture-such as environmental conditions, cytokines, and chemical additives-is vital for producing high-quality therapeutic proteins.

## Data Availability

Not applicable.
